# Measuring Digital Health Literacy in Older Adults: Development and Validation Study

**DOI:** 10.2196/65492

**Published:** 2025-02-05

**Authors:** SungMin Kim, Choonghee Park, Sunghyeon Park, Dai-Jin Kim, Ye-Seul Bae, Jae-Heon Kang, Ji-Won Chun

**Affiliations:** 1 Department of Medical Informatics Seoul St. Mary’s Hospital The Catholic University of Korea College of Medicine Seoul Republic of Korea; 2 Department of Psychiatry Seoul St. Mary’s Hospital The Catholic University of Korea College of Medicine Seoul Republic of Korea; 3 Big Data Research Center School of Medicine Kangbuk Samsung Hospital Sungkyunkwan University Seoul Republic of Korea; 4 Department of Family Medicine Kangbuk Samsung Hospital Sungkyunkwan University School of Medicine Seoul Republic of Korea; 5 The Catholic Medical Center Institute for Basic Medical Science The Catholic Medical Center of The Catholic University of Korea Seoul Republic of Korea

**Keywords:** digital health care, older adults, digital health literacy, exploratory factor analysis, confirmatory factor analysis, mobile phone

## Abstract

**Background:**

New health care services such as smart health care and digital therapeutics have greatly expanded. To effectively use these services, digital health literacy skills, involving the use of digital devices to explore and understand health information, are important. Older adults, requiring consistent health management highlight the need for enhanced digital health literacy skills. To address this issue, it is imperative to develop methods to assess older adults’ digital health literacy levels.

**Objective:**

This study aimed to develop a tool to measure digital health literacy. To this end, it reviewed existing literature to identify the components of digital health literacy, drafted preliminary items, and developed a scale using a representative sample.

**Methods:**

We conducted a primary survey targeting 600 adults aged 55-75 years and performed an exploratory factor analysis on 74 preliminary items. Items with low factor loadings were removed, and their contents were modified to enhance their validity. Then, we conducted a secondary survey with 400 participants to perform exploratory and confirmatory factor analyses.

**Results:**

A digital health literacy scale consisting of 25 items was developed, comprising 4 subfactors: use of digital devices, understanding health information, use and decision regarding health information, and use intention. The model fit indices indicated excellent structural validity (Tucker-Lewis Index=0.924, comparative fit index=0.916, root-mean-square error of approximation=0.088, standardized root-mean-square residual=0.044). High convergent validity (average variance extracted>0.5) and reliability (composite reliability>0.7) were observed within each factor. Discriminant validity was also confirmed as the square root of the average variance extracted was greater than the correlation coefficients between the factors. This scale demonstrates high reliability and excellent structural validity.

**Conclusions:**

This study is a significant first step toward enhancing digital health literacy among older adults by developing an appropriate tool for measuring digital health literacy. We expect this study to contribute to the future provision of tailored education and treatment based on individual literacy levels.

## Introduction

### Background

The proliferation of digital technology and the widespread use of smart devices have facilitated convenient and ubiquitous access to current information and services. The worldwide adoption rate of smartphones is approximately 76%, with South Korea having the highest rate at 95% [1]. The diverse services in the digital realm can immensely benefit individuals using them [2]. Cutting-edge medical services, such as digital therapeutic gadgets, intelligent monitoring, and telemedicine are intricately connected to the digital revolution in health care [3]. The medical device sector, in an attempt to actively identify and meet patient needs, develops digital health care services at medical treatment sites. These digital health care services improve the accessibility of medical care and individualized therapy [4]. For instance, the use of smartphone applications for medication reminders and the implementation of disease monitoring systems that rely on patient-generated health data prove highly beneficial for patients with chronic illnesses [5]. To effectively use digital health care services, patients and those without underlying medical conditions must possess the necessary skills to retrieve and use the desired health information in the digital domain [6,7].

Digital health literacy is a newly developed term that combines 2 distinct concepts: health and digital literacy [8]. Beyond comprehending health information, it includes the ability to efficiently use digital tools and resources for health care. However, the adoption of new technologies varies with the social environment and individual abilities. These disparities contribute to a digital divide and information gap. In South Korea, digitally marginalized groups, including older adults, rural areas residents, individuals with disabilities, and low-income individuals, are unable to keep up with advancements in digital technology [9], with studies recognizing the digital divide as a noteworthy societal problem among these populations [10-12]. The most vulnerable group is the older adult population, who have limited exposure to smart devices and limited opportunities for education in information and communication technology [13]. The COVID-19 pandemic has exacerbated this phenomenon [14].

By 2025, South Korea’s population aged 65 years and older is projected to surpass 20% of the total population [15]. Over the next decade, population aging is expected to worsen, with the proportion of older adults steadily increasing [16]. Consequently, South Korea and other countries with aging populations have recognized digital exclusion among older adults as a new social issue. Older adults experience difficulties using everyday technologies such as kiosks and self-checkout counters and medical services such as digital therapeutic devices and telemedicine [17]. Although social support can motivate older adults to use digital technologies [18], those living alone may have limited access to digital health care services owing to a lack of assistance from family members. Therefore, it is crucial to develop a strategy that enhances the digital health literacy level among those lacking access to digital services. Enhancing digital health literacy can offer significant health benefits for individuals [19]. Individuals using digital health services understand which information is most appropriate for their needs, while service providers can deliver optimum services to them [20]. For instance, wearable devices such as smartwatches can transmit real-time vital health signs data including temperature, heart rate, blood pressure, and stress levels, to health care providers, thereby enabling continuous monitoring of patients’ conditions [21]. To improve digital health literacy, it is essential to assess the current level of digital health literacy among users.

Despite efforts to assess digital health literacy, there has been a deficiency of thorough, comprehensive research that specifically examines and quantifies its various dimensions [22]. According to the previous systematic review investigating the measurement properties of eHealth literacy instruments, the most widely used scale is the eHealth Literacy Scale [23]. This scale estimated the ability to locate, comprehend, evaluate, and use electronic health information to address health concerns [24]. However, this assessment was insufficient in evaluating digital health literacy comprehensively [25], as it was developed before the emergence of social media and mobile-based health care technologies and was reported to not reflect recent advancements in digital health technologies [23]. van der Vaart and Drossaert [26] developed the Digital Health Literacy Instrument to measure various competencies, emphasizing the ability to use internet information. However, their study’s reliance on a highly educated sample might have overestimated digital skills, and the limited platforms used could affect generalizability. Karnoe et al [27] categorized digital health literacy into 7 components, 4 related to health literacy and 3 to digital literacy, and developed a corresponding measurement scale. However, this scale only evaluates the comprehension of health information and the use of digital technologies without considering social literacy elements such as information sharing. Also, the transactional eHealth literacy instrument was evaluated as the best instrument for comprehensive assessments of eHealth literacy; however, its applicability was limited to healthy individuals or patients younger than 40 years [23].

Furthermore, previous studies limited that could not represent certain populations such as older adults or low-income individuals. Age is often cited as a sociodemographic factor that can influence digital health literacy, with evidence generally showing that digital health literacy tends to decline with increasing age [28]. Particularly, the previous study on digital literacy assessment tools for older persons found that further research on the appropriateness of these instruments for this demographic is necessary [29]. This suggests that there may be differences in digital habits and learning potential between older and younger individuals dependent on needs and experiences with digital technology, highlighting the need for specialized measurement tools tailored to the older.

### Objective

This study aimed to identify the fundamental components of digital health literacy among older adults and develop a systematic self-report scale that can be used to effectively assess their level of digital health literacy.

## Methods

### Overview

**Figure 1:**
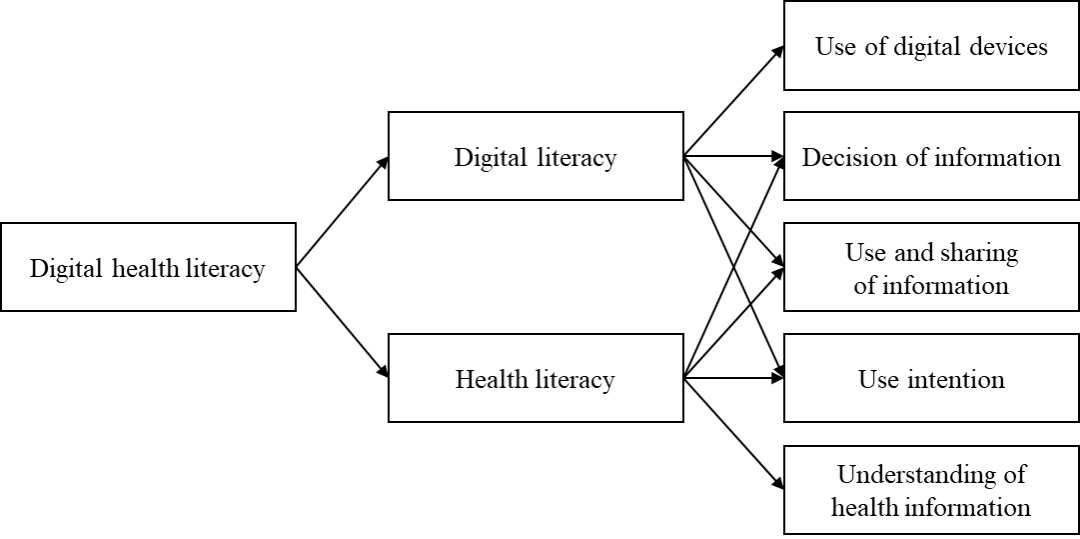
Factor structure of digital health literacy.

This study investigated the subjective experiences of digital health literacy among older adults, focusing on their ability to use digital technologies and skills in searching for, understanding, and using health information (Figure 1). We used 2 approaches to create an item pool: a literature review to identify digital health literacy components and focus interviews with health care experts to validate these items. Subsequently, we used a 2-step survey and analysis using exploratory and confirmatory factor analyses.

### Development of the Digital Health Literacy Scale

We examined previous studies to develop an initial item pool based on pertinent hypotheses regarding motives, functionality, and experiences related to health care technology use among older adults. To achieve this, we searched multiple sources, including the National Library of Medicine PubMed database and Web of Science, using predefined keywords such as “Digital Health Literacy scale,” “Mobile health device,” “eHealth,” “Health Literacy,” and “Digital Literacy.” The inclusion criteria for selecting studies were as follows: (1) studies focusing on digital health literacy or related concepts, (2) studies providing quantitative evaluations of digital health literacy tools, and (3) studies where measurement scales were openly available in the publication or could be obtained upon request. Exclusion criteria included: (1) studies that did not explicitly assess digital health literacy, (2) studies without quantitative data on digital health literacy tools, and (3) studies where measurement scales were unavailable or inaccessible upon request. Two reviewers (SMK and JWC) independently extracted appropriate instruments, considering study design, target population, measured variables, and psychometric properties of the scales, and discrepancies were resolved through consensus. The final selection of items was determined through discussions among the research team, which included medical doctors, medical informatics experts, and psychologists.

The final item pool was informed by a detailed review of five key instruments: (1) the Mobile Device Proficiency Questionnaire (covering basics, communication, data storage, and internet use), (2) European Health Literacy Survey Questionnaire (assessing the ability to understand and process health information), (3) Unified Theory of Acceptance and Use of Technology (evaluating perceived usefulness, ease of use, and attitudes), (4) digital health literacy instrument (focusing on the reliability of health care information), and (5) Transactional eHealth Literacy Instrument (related to health information communication) [27,30-33]. In addition, by reviewing the literature on digital health literacy and older adults [23,29], we categorized validated and available scales, identifying the characteristics of each scale item as well as their limitations. Therefore, the scales used in this study were selected based on two key criteria. First, each scale has been validated for reliability and validity in previous research, ensuring their credibility as assessment tools. Second, the scales were chosen for their ability to comprehensively evaluate various aspects of digital health literacy, such as mobile device proficiency, health information processing, and attitudes toward technology acceptance, allowing for a well-rounded measurement.

Our second approach involved a focus group interview with 10 experts from nursing, social work, public health, education, and medicine. These experts regularly engage with older individuals in digitized medical environments, offering several forms of assistance, which understand the issues and demands typically encountered by seniors. Focus group interviews were conducted with participants in 2 groups of 5, each lasting 2 hours. The interview was audio-recorded, and transcribed immediately after the interview. The data was categorized into 3 overarching themes by the researchers, who independently extracted meaningful units from the transcripts. The initial theme, “Accessibility of Digital Health care Services,” addressed concerns such as the ability to pay medical expenses through hospital applications and the necessity of locating hospital information during emergencies. The second theme, “Understanding and Utilizing Medical Information,” encompassed the capacity to effectively interpret and use health check-up results, medication instructions, and nutritional information. The third theme, “Autonomy through Digital Health Technologies,” underscored the ability to use health-related applications independently to access medical services or evaluate personal health without external assistance. Items derived from these themes were developed and refined through expert feedback to ensure their relevance and were subsequently included in the preliminary scale. The guidelines for focus group interviews are included in Multimedia Appendix 1. The initial item pool included 74 items: 59 from the literature review and 15 from focus group interviews (Multimedia Appendix 2).

### Participants and Data Collection

The target population comprised Korean adults aged 55 years to 75 years. The target population comprised Korean adults aged 55 years to 75 years. Participants were recruited using a closed survey approach in the platform of the survey agency via emails and website visits. Quota sampling was used to ensure balanced representation by gender and age groups. Data collection was conducted in two phases: the first survey included 600 participants from August to September 2023, and the second survey included 400 participants from October to November 2023. Individuals who participated in the first survey were not eligible to participate in the second survey. To prevent duplicate responses, each participant accessed the survey through a unique URL. IP address checks and cookie-based mechanisms were implemented to ensure data quality and prevent multiple submissions from the same individual.

### Data Analysis

#### Exploratory Factor Analysis

To conduct exploratory factor analysis (EFA), an absolute minimum of 100 participants is required, with 200 participants considered fair and 300 participants deemed good [34,35]. With 600 participants, we performed the first EFA to explore the scale’s factor structure and selected items using IBM SPSS Statistics for Windows (version 25.0). Moreover, we conducted frequency analysis to verify demographic information and reliability analysis. Descriptive statistics are summarized as frequencies, percentages, or means (SD). The factor extraction method used was the maximum likelihood method with direct oblimin rotation to calculate factor loadings [36]. We present the number of factors to 5 based on the literature review, which was verified using parallel analysis [37]. To ensure the item structure’s validity, we followed factor analysis guidelines, removing items with factor loadings below 0.4 and items with cross-loadings above 0.3 on 2 or more factors [38]. The adequacy of the factor analysis was assessed using the Kaiser-Meyer-Olkin (KMO) measure and Bartlett’s test of sphericity.

In addition, we conducted a second EFA with a sample of 200 randomly selected individuals from the entire pool of 400 participants in the second survey to validate the final 33 items in the first survey. A determining factor is the ratio of tested items to participants, with a ratio of 5 participants per item considered appropriate [35]. The EFA was conducted using the same method as in the first survey. As before, items with factor loadings below 0.4 and items with cross-loadings above 0.3 on 2 or more factors were removed to select the final items for the confirmatory factor analysis (CFA).

#### CFA to Validate the Scale’s Structure

To validate the scale structure, which consisted of 25 items, we conducted a CFA with 200 participants after the second EFA using IBM SPSS Amos for Windows (version 25.0), and structural equation modeling. It is known that the minimum sample size for CFA is to have at least 200 participants [39] and a ratio of sample size to the number of model parameters is more than 5 in factor analysis [35]. Model fit was evaluated using the chi-square statistic, comparative fit index (CFI), Tucker-Lewis Index (TLI), standardized root-mean-square residual (SRMR), and root-mean-square error of approximation (RMSEA). A model with the ratio of the chi-square statistic to the respective df less than 3, CFI and TLI values greater than 0.90, and SRMR and RMSEA less than 0.08 was considered an acceptable fit. In addition, convergent validity was assessed using average variance extracted (AVE) values, ensuring that they were above 0.5, and construct reliability (CR) with values above 0.7. Discriminant validity was evaluated by comparing the square root of the AVE for each construct with the correlations between the constructs [40].

### Ethical Considerations

This study received approval from the institutional review board of the Catholic University of Korea, Songeui Campus (approval number: MIRB 20230825-007). The study adhered to institutional and national ethical guidelines for research involving human participants. Before participation, all participants received detailed information about the study’s purpose, procedures, anonymous, data usage, and their rights. Participation was voluntary, and participants could withdraw at any point. Data protection measures were implemented, including the anonymization of all collected data to prevent unauthorized access. No personal identifiers such as names or contact information were collected. Only completed surveys were included in the final analysis. The survey was web-based and conducted through the online platform of the survey agency (Macromill Embrain Co. Ltd.). Participants who completed the survey received a reward of 500 points (approximately US $0.35) redeemable within the online platform.

## Results

### Participants

The mean age of the sample was 63.63 (SD 5.26) years, and 50% (500/1000) were women. Further, 90.8% (908/1,000) were married, and 4.0% (40/1,000) were single. Regarding the presence of chronic diseases, 48.2% (428/1,000) had been diagnosed with at least one of hypertension, diabetes, or hyperlipidemia, while 51.8% (518/1,000) had not been diagnosed with any chronic disease. Regarding subjective economic status, 51.5% (515/1,000) and 48.5% (485/1,000) indicated that their economic position was above average and below average, respectively. Regarding subjective health status, 87.5% (875/1,000) and 12.5% (125/1,000) reported their health to be above average and below average, respectively. Participants provided demographic data encompassing gender, age, marital status, chronic disease, subjective economic situation, and subjective health status, along with digital health literacy items (Table 1).

**Table 1:** The demographic characteristics (N=1000).

Characteristics			First survey (n=600), n (%)		Second survey (n=400), n (%)		Participants, n (%)
**Sex**							
	Male	300 (50)		200 (50)		500 (50)	
	Female	300 (50)		200 (50)		500 (50)	
**Age (years)**							
	55-65	300 (50)		200 (50)		500 (50)	
	66-74	300 (50)		200 (50)		500 (50)	
**Marital status**							
	Married	557 (92.8)		351 (87.8)		908 (90.8)	
	Not married	17 (2.8)		23 (5.9)		40 (4)	
	Divorced	26 (4.4)		26 (6.5)		52 (5.2)	
**Chronic disease (hypertension, diabetes, or hyperlipidemia)**							
	Yes	276 (46)		206 (51.5)		482 (48.2)	
	No	324 (54)		194 (48.5)		518 (51.8)	
**Subjective economic level**							
	High	3 (0.5)		3 (0.8)		6 (0.6)	
	Upper middle	50 (8.3)		37 (9.3)		87 (8.7)	
	Middle	261 (43.5)		161 (40.3)		422 (42.2)	
	Low middle	233 (38.8)		166 (41.5)		399 (39.9)	
	Low	53 (8.8)		33 (8.3)		86 (8.6)	
**Subjective health status**							
	Very good	21 (3.5)		12 (1)		33 (3.3)	
	Good	233 (38.8)		127 (11.8)		360 (36)	
	Average	272 (45.3)		210 (52.5)		482 (48.2)	
	Not good	72 (12)		47 (31.8)		119 (11.9)	
	Not very good	2 (0.3)		4 (3)		6 (0.6)	

### First Validation Survey: EFA

Based on the literature review and focus group interviews (Multimedia Appendix 3), we assembled a pool of 74 items reflecting the 5 factors of older adults’ digital health literacy and extracted the underlying structure of items using EFA. In the first survey sample (n=600), all participants were aged 55-75 years, and 50% (n=300) were women. The number of factors was determined using parallel analysis. The eigenvalues of the real data were more significant than those of the random data up to the fourth eigenvalue (real data eigenvalue=0.461; random data eigenvalue=0.334). However, from the fifth eigenvalue onwards, the eigenvalues of the real data were smaller than those of the random data (real data eigenvalue=0.160; random data eigenvalue=0.299). Following the examination of item discrimination and determination of factor loadings, 29 items were retained. The domains of health information decision and use and sharing were consolidated, while other aspects remained. The KMO value, representing the suitability of factor analysis, was 0.981, and Bartlett’s test of sphericity yielded a significant result (*χ*²_406_=19365.0, *P*<.001), confirming the appropriateness of the model. The results also demonstrated a satisfactory level of internal consistency for each factor (Cronbach α values: 0.925-0.944). Table 2 lists the selected components’ commonalities and factor loadings.

**Table 2:** Exploratory factor analysis results for survey 1.

Factors and Items		Communality	Standard Factor Loading
**Understanding of Health Information**			
	Q42. I can understand the instructions for medication provided by the hospital app.	0.861	0.902
	Q41. I can understand the emergency manual provided by health-related apps.	0.824	0.770
	Q39. I can understand the health check-up results provided by the hospital app.	0.794	0.752
	Q48. I can understand the terms of the privacy consent form when registering on the hospital app.	0.781	0.703
	Q40. I can understand the nutritional information of food provided by health-related apps.	0.803	0.700
	Q49. I am aware of the precautions for online payment when paying medical bills.	0.772	0.636
	Q46. I can understand the information about health check-ups (such as target, date, price, fasting requirements, etc.) provided by the hospital app.	0.811	0.566
**Use of Digital Devices**			
	Q7. I can save photos and texts about healthy activities found on the Internet.	0.765	0.856
	Q9. I can use the desired services (payment, location search, etc.) through the hospital kiosk.	0.709	0.851
	Q13. I can delete health-related apps that I have used.	0.737	0.839
	Q8. I can book and confirm medical services through the hospital app.	0.745	0.801
	Q6. I can find a suitable hospital for my symptoms using my smartphone.	0.723	0.792
	Q3. I can find information about disease symptoms and treatments using my smartphone.	0.661	0.770
	Q12. I can use the online “store” (e.g., Apple App Store or Google Play Store) on my device to find health-related apps.	0.711	0.764
	Q15. I can use appropriate words or search terms to find the health service information I want on the Internet.	0.788	0.716
	Q14. I can access hospital websites through an Internet search.	0.793	0.714
	Q17. In an emergency, I can find information about nearby hospitals using my smartphone.	0.751	0.678
**Use Intention**			
	Q32. I believe it is necessary to exchange health information online.	0.692	0.885
	Q33. I am willing to use health-related apps to collect health information.	0.753	0827
	Q30. I have a lot of interest in health-related apps.	0.678	0.790
	Q35. I am interested in learning health knowledge or skills from the Internet.	0.758	0.739
	Q29. I find the necessity and convenience of health management through health-related apps.	0.682	0.507
	Q25. Using health-related apps improves my ability to manage my health.	0.580	0.431
**Use and Decision of Health Information**			
	Q57. I can judge whether the health information found on my smartphone is trustworthy.	0.784	0.886
	Q58. I can evaluate the pros and cons of various treatment methods provided by the hospital app.	0.771	0.785
	Q61. I can determine the medical services I need.	0.734	0.707
	Q59. I can judge how to use the health information provided by health-related apps.	0.753	0.705
	Q51. I can determine if the health information found on the Internet is written for commercial purposes (advertisements).	0.706	0.516
	Q64. I can use the health information provided by health-related apps for disease management.	0.774	0.449

In addition, we made minor adjustments to the working scale to align it with the conceptual framework of digital health literacy. First, the health information decision and use and sharing were combined to create a new factor called the use and decision of health information. Second, hospital apps were renamed health-related apps to encompass a broader range of applications. Finally, 4 new items related to digital health services were added. As a result, we created 33 items divided into 4 domains and their associated subscales (Multimedia Appendix 4).

### Second Validation Survey: EFA

We randomly selected 200 responses (women 53.0%) from the 400 valid and unique responses in the second survey. Majority of the respondents were aged 55-65 years (51.5%). Of the 200 participants, 86.5% (173/200) were married, 4% (8/200) unmarried, and 9.5% (19/200) divorced. The subjective economic level was categorized as high (2/200, 1%), upper middle (21/200, 10.5%), low middle (85/200, 42.5%), or low (16/200, 8.0%). Subjective health status was categorized into four groups: (1) high (4/200, 2.0%), (2) upper middle (62/200, 31.0%), (3) low middle (21/200, 10.5%), and (4) low (1/200, 0.5%).

The results of the secondary EFA also supported the feasibility of performing factor analysis (KMO 0.945; Bartlett test of sphericity: *χ*²_300_=4664.2, *P*<.001). Parallel analysis indicated that the 4 factors were consistent with the primary study. The fifth eigenvalue for the real data was 0.207, while that for the random data was 0.320. The internal reliability of each component and subscale was satisfactory, as indicated by the Cronbach α values for the 4 subscales falling between 0.91 and 0.96, which is above the minimum threshold of 0.7. The first factor (use of digital devices) comprised 10 items and had a Cronbach α value of 0.96. The second factor (understanding health information) had 5 items and exhibited a reliability coefficient of .92. The third (use and decision of health information) and fourth factors (use intention) consisted of 5 items and demonstrated high reliability, with coefficients of 0.91 and 0.93, respectively. The 25-item scale demonstrated a high level of dependability with a value of 0.96. Almost all items had factor loadings above 0.6 (Table 3). Overall, these results suggest that the 4 factors extracted through EFA were appropriate.

**Table 3:** Exploratory factor analysis results for survey 2.

Factors and items		Communality	Standard factor loading
**Use of digital devices**			
	Q15. I can access the hospital’s website through an internet search.	0.757	0.911
	Q30. I can register as a member on the hospital website or app.	0.697	0.841
	Q11. I can delete health-related apps that I have used.	0.636	0.828
	Q17. In an emergency, I can find information about nearby hospitals using my smartphone.	0.762	0.788
	Q33. I can make online payments for medical bills through the hospital website or app.	0.745	0.774
	Q12. I can use the online “store” (eg, Play Store or App Store) on my device to find health-related apps.	0.724	0.774
	Q16. I can use appropriate words or search terms to find the health service information I want on the internet.	0.697	0.741
	Q8. I can use the desired services (payment, location search, etc.) through the hospital kiosk.	0.628	0.739
	Q9. I can book and confirm medical services through the hospital app.	0.716	0.696
	Q31. I can find my test results or prescription details on the hospital website or app.	0.633	0.590
**Understanding of health information**			
	Q1. I can understand the instructions for medication provided by health-related apps.	0.775	0.903
	Q3. I can understand the emergency manual provided by health-related apps.	0.737	0.775
	Q5. I can understand the nutritional information of food provided by health-related apps.	0.629	0.767
	Q7. I can understand the information about health check-ups (such as target, date, price, fasting requirements, etc.) provided by health-related apps.	0.765	0.756
	Q4. I can understand the health check-up results provided by health-related apps.	0.594	0.636
**Use and decision of health information**			
	Q24. I can judge whether the health information found on my smartphone is trustworthy.	0.790	0.836
	Q25. I can evaluate the pros and cons of various treatment methods provided by health-related apps.	0.719	0771
	Q29. I can determine if the health information found on the Internet is written for commercial purposes (advertisements).	0.607	0.681
	Q26. I can judge how to use the health information provided by health-related apps.	0.700	0.609
	Q27. I can determine the medical services I need.	0.611	0.570
**Use intention**			
	Q18. I believe it is necessary to exchange health information online.	0.794	0.956
	Q21. I have a lot of interest in health-related apps.	0.812	0.885
	Q19. I am willing to use health-related apps to collect health information.	0.795	0.859
	Q22. I find the necessity and convenience of health management through health-related apps.	0.734	0.761
	Q23. Using health-related apps improves my ability to manage my health.	0.638	0.619

### CFA

We performed CFA on a sample of 200 responses (women 47.0%) that were not included in the previous EFA from the 400 valid and unique responses in the second survey. Majority of respondents were aged 66-74 years, comprising 103 of 200 (51.5%) respondents. Of the 200 participants, 89% (178/200) were married, 7.5% (15/200) unmarried, and 3.5% (7/200) divorced. Subjective economic level was classified into four categories: (1) high (1/200, 0.5%), (2) upper middle (16/200, 8.0%), (3) low middle (81/200, 40.5%), and (4) low (17/200, 8.5%). Subjective health condition was classified into four categories: (1) high (8/200, 4.0%), (2) upper middle (65/200, 32.5%), (3) low middle (26/200, 13.0%), and (4) low (3/200, 1.5%). Following the outcomes of the previous EFA, 4 distinct factors were identified, and all 25 items were associated with each component. The model demonstrated strong fit across key indices. The CFI was 0.924, and the TLI was 0.916, both surpassing the commonly accepted threshold of 0.90, indicating a good fit. The SRMR was 0.044, reinforcing the adequacy of the model. The RMSEA was 0.088, with a 90% CI between 0.080 and 0.096, falling within acceptable limits. These results confirm the robustness of the 4-factor structure and its alignment with the theoretical framework.

**Figure 2:**
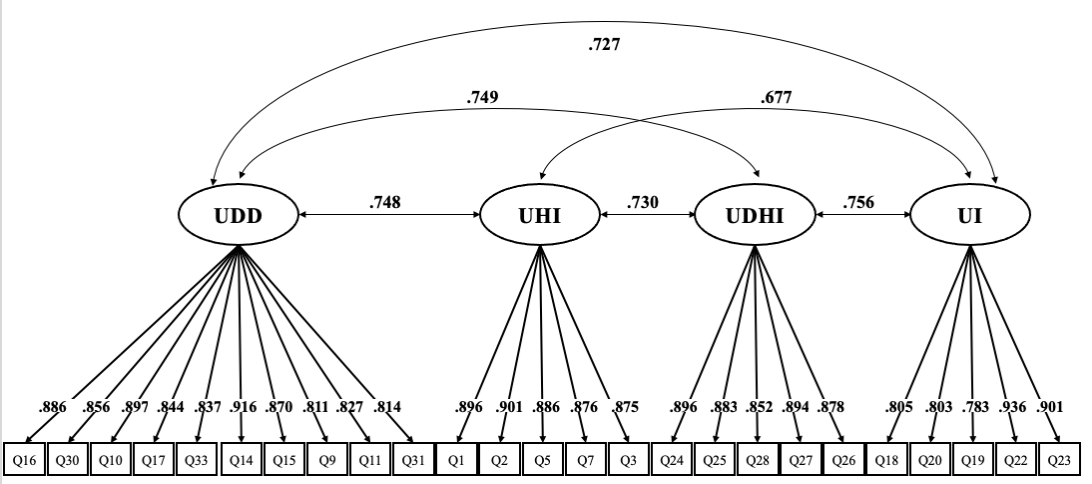
Factor structure with standardized loading for Korean version of the digital health literacy Scale. UDD: use of digital devices; UHI: understanding health information; UDHI: use and decision of health information; UI: use intention.

The factor loadings per item in Table 4 were all greater than 0.7, indicating a strong relationship between the items and their respective factors. The AVE values were higher than 0.7 and CR values ranged from 0.93 to 0.96. The high AVE and CR values of this scale indicate that it adequately explains the latent construct it aims to measure and demonstrates high internal consistency. Therefore, the results support the convergent validity. Discriminant validity was also confirmed, as the square roots of the AVE values for each factor were greater than the correlations between the factors. Figure 2 displays the factor structure and standardized factor loadings obtained from the CFA, which demonstrated the validity of the scale developed in this study. The final scale comprised 4 factors and 25 items (Multimedia Appendix 5). Participants assign 4 points for strong agreement and 0 points for severe disagreement to each item. The digital device use component consists of 10 items, contributing up to 40 points. The remaining three components which are (1) understanding health information, (2) use and decision-making of health information, and (3) use intention, each consist of 5 items, contributing up to 20 points per component.

**Table 4:** Confirmatory factor analysis results for survey 2.

Factors and items		Standard factor loading	Squared multiple correlations	AVE^a^		CR^b^	
**Use of digital devices**					0.734		0.965
	Q16. I can access the hospital’s website through an Internet search.	0.886	0.78				
	Q30. I can register as a member on the hospital website or app.	0.856	0.73				
	Q10. I can delete health-related apps that I have used.	0.897	0.80				
	Q17. In an emergency, I can find information about nearby hospitals using my smartphone.	0.844	0.71				
	Q33. I can make online payments for medical bills through the hospital website or app.	0.837	0.70				
	Q14. I can use the online “store” (eg, Play Store or App Store) on my device to find health-related apps.	0.916	0.84				
	Q15. I can use appropriate words or search terms to find the health service information I want on the internet.	0.870	0.76				
	Q9. I can use the desired services (payment, location search, etc.) through the hospital kiosk.	0.811	0.66				
	Q11. I can book and confirm medical services through the hospital app.	0.827	0.68				
	Q31. I can find my test results or prescription details on the hospital website or app.	0.814	0.66				
**Understanding of health information**					0.787		0.949
	Q1. I can understand the instructions for medication provided by health-related apps.	0.896	0.80				
	Q2. I can understand the emergency manual provided by health-related apps.	0.901	0.81				
	Q5. I can understand the nutritional information of food provided by health-related apps.	0.886	0.78				
	Q7. I can understand the information about health check-ups (such as target, date, price, fasting requirements, etc.) provided by health-related apps.	0.876	0.77				
	Q3. I can understand the health check-up results provided by health-related apps.	0.875	0.77				
**Use and decision of health information**					0.776		0.945
	Q24. I can judge whether the health information found on my smartphone is trustworthy.	0.896	0.80				
	Q25. I can evaluate the pros and cons of various treatment methods provided by health-related apps.	0.883	0.78				
	Q28. I can determine if the health information found on the internet is written for commercial purposes (advertisements).	0.852	0.73				
	Q27. I can judge how to use the health information provided by health-related apps.	0.894	0.80				
	Q26. I can determine the medical services I need.	0.878	0.77				
**Use Intention**					0.719		0.927
	Q18. I believe it is necessary to exchange health information online.	0.805	0.65				
	Q20. I have a lot of interest in health-related apps.	0.803	0.64				
	Q19. I am willing to use health-related apps to collect health information.	0.783	0.61				
	Q22. I find the necessity and convenience of health management through health-related apps.	0.936	0.88				
	Q23. Using health-related apps improves my ability to manage my health.	0.901	0.81				

^a^AVE: average variance extracted.

^b^CR: construct reliability.

## Discussion

### Principal Results

This study aimed to develop and validate a scale for measuring digital health literacy. The only validated digital health literacy scale previously translated and validated in Korea was the eHealth Literacy Scale adapted by Chang et al [41]. Despite its extensive translation and use in many countries [42], this scale does not keep pace with current technological development. We developed a Korean version of the Digital Health Literacy Scale designed specifically for older adults who are vulnerable to the digital information divide. This scale has the distinctive feature of being specifically designed for older adults, who are an important but often overlooked population in digital health literacy research. This scale comprises 25 items across 4 main factors: use of digital devices, processing of health information, use and reliability assessment of health information, and evaluation of usage intentions. While other scales primarily focus on the general population or specific health conditions, our scale addresses the unique challenges faced by older adults, such as lower familiarity with technology and specific health information needs. The Korean version of the digital health literacy scale considers various factors, making it a valuable tool for comprehensively assessing the digital health literacy level of older adults. Furthermore, it can serve as an essential foundational resource for developing digital health literacy education programs and applying patient-tailored digital therapeutic devices for older adults.

Use of Digital Devices evaluates the ability to search for and use health information using digital devices such as smartphones, tablets, and computers. Efficient digital device usage is essential for searching and using health information, making it a fundamental element of digital health literacy. Studies have shown that older adults have lower skill levels in using the internet to search for health information compared to younger people [43], with a lack of digital device proficiency cited as a primary cause. Therefore, assessing the use of digital devices on this scale can help develop tailored strategies for enhancing older adults’ digital health literacy capabilities.

Understanding of Health Information measures the ability to understand and practically apply health information. Although many applications are available to assist individuals in health management, if users fail to comprehend basic health information, they will struggle to use these tools effectively, resulting in poor health management. Accurately measuring the ability to understand and apply health information based on this factor can serve as a starting point for improving digital health literacy.

Use and Decision of Health Information measures the ability to evaluate the reliability of health information obtained through digital devices and apply it in practice. This is a crucial aspect of digital health literacy, as it includes the ability to use reliable information to engage with personal health information. For instance, it includes the ability to determine whether health information found on the Internet is for commercial purposes. Initially, we hypothesized that “Decision of Health Information” and “Utilization and sharing of Health Information” would form separate factors. However, it revealed that combining these items into a single factor was a more suitable structure that improves the internal consistency of the factor and strengthens its interpretability. Consequently, the results indicated that older persons viewed behaviors associated with decision-making, using, and sharing health information as a cohesive behavioral pattern rather than as separate processes. Compared to younger people, older adults might find it more challenging to discern false information [44], which increases the risk of accepting incorrect health information. Given the vast amount of information available online, it is essential to identify the relevant information and that which contains evidence. Particularly, it is necessary to include items such as digital content creation and digital safety [29]. The questions measuring digital content creation and digital safety were included in the initial items (Multimedia Appendix 2). These items were excluded to enhance the reliability and validity of the scale, but future studies should focus on developing and validating questions that can assess these aspects.

Finally, Use Intention measures an individual’s intention to use digital health applications, that is the motivation and willingness to adopt and use new technologies, which are critical elements for enhancing an individual's digital health literacy level. One of the reasons older adults do not use digital devices is a negative attitude toward new technologies, such as a sense of resistance [45]. Consequently, older adults are slower to adopt new technologies, and even when educational programs are necessary, they may exhibit low learning motivation. For individuals with low intention to use digital health devices, it is critical to first raise awareness of the importance of digital health literacy and present convenient health services. This, in turn, will strengthen digital health literacy.

Digital health literacy significantly improves older adults’ ability to manage their health and the effectiveness of digital health care services. This study highlights the importance of digital health literacy in South Korea’s medical domain and provides fundamental data for addressing digital exclusion among older adults. Moreover, this study used a distinct approach to assess the digital health literacy of older adults using the 4 key factors, distinguishing it from previous studies. This approach contributes to a more systematic understanding of digital health literacy and a more precise assessment of older adults’ actual literacy levels. By measuring overall digital device proficiency as well as the ability to understand and use health information, this scale can be effectively used in countries where smartphones are widely available. In the future, research is needed not only to validate this scale across diverse cultural contexts to enhance its generalizability but also to evaluate its predictive validity, thereby ensuring its utility in identifying individuals' properties with low digital health literacy.

### Limitations

First, because data were collected through an online survey, it is possible that participation from older adults unfamiliar with digital devices was limited and resulted in an underestimation of the actual digital health literacy levels in groups with low literacy. Therefore, future studies should consider parallel offline surveys for more accurate data. Second, this study focused on older adults, who are the most vulnerable to digital exclusion [13], which may limit the findings’ generalization to other age groups or populations with diverse backgrounds. To achieve broader generalizability, future research should aim to collect samples that include a wider range of ages and backgrounds. Third, we prioritized expert feedback during the item development stage to ensure content validity but did not directly include focus group interviews with older participants to gather insights into their digital device usage for health management. Future studies should consider conducting focus group interviews with older adults to further refine the scale and enhance its relevance to this population. Finally, due to the disparity in total scores among the subscales, caution should be observed while using the overall score.

### Conclusions

In conclusion, this study developed and validated a novel scale to assess digital health literacy among older adults, addressing the unique challenges posed by the digital information divide in this population. The Digital Health Literacy Scale, comprising 25 items across four factors, provides a comprehensive framework for evaluating older adults’ digital device proficiency, understanding of health information, decision-making, and usage intentions. By highlighting these dimensions, the scale facilitates tailored interventions to enhance digital health literacy, ultimately improving health management outcomes and the adoption of digital healthcare services. This study underscores the necessity of targeted approaches in promoting digital inclusion for older adults, especially in rapidly digitizing societies. Future research should extend this work by validating the scale across diverse cultural and demographic contexts and exploring its utility in predicting health-related behaviors and outcomes.

## Appendix

Multimedia Appendix 1 Focus group interview guidelines for developing a digital health literacy scale.

Multimedia Appendix 2 Preliminary Items for Digital Health Literacy Scale. 5-point Likert-scale. 0–4 (Strongly Disagree-Strongly Agree).

Multimedia Appendix 3 PRISMA 2020 Checklist.

Multimedia Appendix 4 Digital Health Literacy Scale items after first exploratory factor analysis. 5point Likert-scale. 0-4(Strongly Disagree-Strongly Agree).

Multimedia Appendix 5 Digital Health Literacy Scale items after confirmatory factor analysis. 5-point Likert-scale. 0–4 (Strongly Disagree-Strongly Agree).

## Abbreviations

AVE: average variance extracted
CFA: confirmatory factor analysis
CFI: comparative fit index
CR: construct reliability
EFA: exploratory factor analysis
KMO: Kaiser-Meyer-Olkin
RMSEA: root-mean-square error of approximation
SRMR: standardized root-mean-square residual
TLI: Tucker-Lewis index
