# Supplementary material for: Measuring Digital Health Literacy in Older Adults: Development and Validation Study
Source: J Med Internet Res. 2025 Feb 5;27:e65492. doi: 10.2196/65492 (PMC11840366; doi:10.2196/65492)
Supplement: Multimedia Appendix 1 [file jmir_v27i1e65492_app1.docx]

Appendix 1. Focus group interview guidelines for developing a digital health literacy scale

1) Defining a focus group

- The focus group interview was conducted to verify and refine items related to older adults’ digital health literacy. By involving experts from various fields who regularly interact with elderly individuals in real-world settings, the research aimed to ensure that the resulting measures would more accurately capture the genuine challenges and needs faced by seniors.
- Participants: 10 participants (divided into two groups of 5 participants each)
- Recruitment Criteria:

a. Healthcare institution employees
b. Individuals performing roles that involve providing services to visitors such as patients at healthcare institutions
c. Adults aged 19 to 74 years old

2) Designing focus group questions

| Questions | |
| --- | --- |
| **Opening questions** | |
|  | "Please feel free to introduce yourselves, including your name and what you are currently doing." |
| **Introduction questions** | |
|  | "Could you share your experiences of encountering digitally vulnerable groups, such as older adults, in your work?"  “How difficult do you think the content of the survey conducted prior to this focus group interview would be for digitally vulnerable groups?" |
| **Transition questions** | |
|  | “Digital health literacy refers to the ability to acquire, understand, and use health information through digital devices. Could you share your own level of digital health literacy and whether you have encountered older adults struggling due to low digital health literacy?” |
| **Key questions** | |
|  | "What do you think about the usability of digital health devices, such as hospital kiosks, for digitally vulnerable groups like older adults?"  "What aspects do you think are the most challenging for digitally vulnerable groups when using the internet?"  "What specific difficulties do digitally vulnerable groups, such as older adults, face during outpatient consultations?"  "What skills and support do you think are most necessary for individuals to proficiently use digital devices?"  "While hospital apps offer many benefits, what do you think prevents some people from using them?"  "Do you believe digital health devices provide convenience to users?"  "What topics and content do you think should be included to enhance the digital health literacy of digitally vulnerable groups?" |
| **Ending questions** | |
|  | "Lastly, is there anything you wanted to say but didn’t get the chance to, or any advice you would like to share with us?" |

3) Recruiting and preparing for participants

- Recruitment advertisements were posted on internal bulletin boards to recruit participants interested in the study.
- After confirming eligibility, participants were grouped into two sets of five in the order they were recruited.
- A researcher assigned to the project conducted the focus group interview and prepared an assistant researcher to record and document participants' responses.

4) Conducting the focus group

- Study participants engaged in a two-hour group interview held in a meeting room.
- The focus group discussion began after participants were informed of its purpose and signed the consent form.
- The demographic information of participants, such as gender, date of birth, job/occupation, and years of experience in healthcare institutions, was collected before the interview.
- The researcher conducted the interview by sequentially asking participants pre-structured questions and managing the speaking time for each participant.
- The discussions were audio-recorded, transcribed after interview immediately.
- Time duration per item

| Question items | Estimated duration |
| --- | --- |
| Opening questions | 2 minutes per each person (Total 10 minutes) |
| Introduction questions | 2 minutes per each person (Total 10 minutes) |
| Transition questions | 2 minutes per each person (Total 10 minutes) |
| Key questions | 15 minutes per each person (Total 75 minutes) |
| Ending questions | 1 minutes per each person (Total 5 minutes) |

5) Analyzing the data

- The data was categorized into three overarching themes by the researchers, who independently extracted meaningful units from the transcripts.
- The initial theme, "Accessibility of Digital Healthcare Services," addressed concerns such as the ability to pay medical expenses through hospital applications and the necessity of locating hospital information during emergencies.
- The second theme, "Understanding and Utilizing Medical Information," encompassed the capacity to effectively interpret and utilize health check-up results, medication instructions, and nutritional information.
- The third theme, "Autonomy through Digital Health Technologies," underscored the ability to utilize health-related applications independently to access medical services or evaluate personal health without external assistance.
