# Supplementary material for: Measuring Digital Health Literacy in Older Adults: Development and Validation Study
Source: J Med Internet Res. 2025 Feb 5;27:e65492. doi: 10.2196/65492 (PMC11840366; doi:10.2196/65492)
Supplement: Multimedia Appendix 2 [file jmir_v27i1e65492_app2.docx]

Appendix 2. Preliminary Items for Digital Health Literacy Scale. 5-point Likert-scale. 0–4 (Strongly Disagree-Strongly Agree)

| Items | |
| --- | --- |
| **Utilization of Digital Devices** | |
|  | 1. If I have abnormal symptoms and signs, I leave an inquiry on the hospital's website. |
|  | 2. I can pay medical bills through the hospital app. |
|  | 3. I can find information about disease symptoms and treatments using my smartphone. |
|  | 4. In an emergency, I can find the necessary first aid methods through the Internet. |
|  | 5. I often get help from others when searching for health information on my smartphone. ® |
|  | 6. I can find a suitable hospital for my symptoms using my smartphone. |
|  | 7. I can save photos and texts about healthy activities found on the Internet. |
|  | 8. I can book and confirm medical services through the hospital app. |
|  | 9. I can use the desired services (payment, location search, etc.) through the hospital kiosk. |
|  | 10. I can obtain medical records through the Internet. |
|  | 11. I can find suitable healthcare professionals through the hospital app. |
|  | 12. I can use the online "store" (e.g., Apple App Store or Google Play Store) on my device to find health-related apps. |
|  | 13. I can delete health-related apps that I have used. |
|  | 14. I can access the hospital website through an Internet search. |
|  | 15. I can use appropriate words or search terms to find the health service information I want on the Internet. |
|  | 16. I do not know where to find information when using health services. ® |
|  | 17. In an emergency, I can find information about nearby hospitals using my smartphone. |
|  | 18. I can find ways to manage unhealthy behaviors (mental health issues) and prevent diseases using my smartphone. |
|  | 19. I can regularly watch educational videos provided by the hospital app. |
| **Use Intention** | |
|  | 20. It is easy for me to obtain health information through health-related apps. |
|  | 21. Learning how to use health-related apps is easy for me. |
|  | 22. Health-related apps are too complicated to use compared to analog methods (e.g., books). |
|  | 23. It takes too much time to learn how to use health-related apps, and it is not worth the effort. |
|  | 24. If I experience difficulties using health-related apps, I give up quickly. |
|  | 25. Using health-related apps improves my ability to manage my health. |
|  | 26. Using health-related apps allows me to use medical services without help from others. |
|  | 27. Using the hospital app makes scheduling appointments faster. |
|  | 28. Using the hospital app makes it easy to share medical details with family and friends. |
|  | 29. I find the necessity and convenience of health management through health-related apps. |
|  | 30. I have a lot of interest in health-related apps. |
|  | 31. I use health-related apps regularly. |
|  | 32. I believe it is necessary to exchange health information online. |
|  | 33. I am willing to use health-related apps to collect health information. |
|  | 34. I can take good care of my health even without using health-related apps. |
|  | 35. I am interested in learning health knowledge or skills from the Internet. |
| **Understanding of Health Information** | |
|  | 36. I can compare various websites to ensure they provide the same health information. |
|  | 37. I can easily follow simple exercise methods provided by health-related apps. |
|  | 38. I have difficulty understanding the terminology used in health information. ® |
|  | 39. I can understand the health check-up results provided by the hospital app. |
|  | 40. I can understand the nutritional information of food provided by health-related apps. |
|  | 41. I can understand the emergency manual provided by health-related apps. |
|  | 42. I can understand the instructions for medication provided by the hospital app. |
|  | 43. I can understand mental health promotion methods provided by health-related apps. |
|  | 44. I know what services are available on the hospital's website. |
|  | 45. I can understand the health warning signs related to excessive drinking, smoking, and lack of exercise provided by health-related apps. |
|  | 46. I can understand the information about health check-ups (such as target, date, price, fasting requirements, etc.) provided by the hospital app. |
|  | 47. I can follow the instructions for medication provided by the hospital app. |
|  | 48. I can understand the terms of the privacy consent form when registering on the hospital app. |
|  | 49. I am aware of the precautions for online payment when paying medical bills. |
| **Decision of Health Information** | |
|  | 50. I can select the necessary information from the health information provided by health-related apps. |
|  | 51. I can determine if the health information found on the Internet is written for commercial purposes (advertisements). |
|  | 52. I can judge whether the health information obtained from health-related apps is applicable to me. |
|  | 53. I can assess my health condition based on the information provided by health-related apps. |
|  | 54. I sometimes find it difficult to select the most relevant health information. ® |
|  | 55. I sometimes feel overwhelmed by the large amount of health information available on the Internet. ® |
|  | 56. It is important to verify health information found on apps, blogs, etc., with professional healthcare providers. |
|  | 57. I can judge whether the health information found on my smartphone is trustworthy. |
|  | 58. I can evaluate the pros and cons of various treatment methods provided by the hospital app. |
|  | 59. I can judge how to use the health information provided by health-related apps. |
|  | 60. I can determine which lifestyle habits (exercise, smoking) need to be modified or improved based on the health information provided by health-related apps. |
|  | 61. I can determine which dietary habits need to be modified or improved based on the dietary information provided by health-related apps. |
|  | 62. I can determine the medical services I need. |
|  | 63. I can determine when to visit the hospital based on the self-health checks provided by health-related apps. |
|  | 64. I can assess my condition based on the health warning signs related to excessive drinking, smoking, and lack of exercise found on the Internet. |
| **Utilization of Health Information** | |
|  | 65. I can utilize the health information provided by health-related apps for disease management. |
|  | 66. I can transfer mental health information (stress and depression management methods) from one phone to another. |
|  | 67. I discuss health-related apps with friends/colleagues. |
|  | 68. I can achieve my health goals (e.g., walking 10,000 steps a day) through health-related apps. |
|  | 69. I can understand the nutritional content provided by health-related apps and create a diet that suits me. |
|  | 70. I can exercise by referring to the exercise methods provided by health-related apps. |
|  | 71. I can share reviews about medical services with others. |
|  | 72. I can write informational posts about disease prevention through blogs, etc. |
|  | 73. I take care to protect personal information when searching for or sharing health information online. |
|  | 74. Based on the nutritional information provided by health-related apps, I eat foods low in fat, saturated fat, and cholesterol. |
