# Supplementary material for: Measuring Digital Health Literacy in Older Adults: Development and Validation Study
Source: J Med Internet Res. 2025 Feb 5;27:e65492. doi: 10.2196/65492 (PMC11840366; doi:10.2196/65492)
Supplement: Multimedia Appendix 4 [file jmir_v27i1e65492_app4.docx]

Appendix 4. Digital Health Literacy Scale items after first exploratory factor analysis. 5point Likert-scale. 0-4(Strongly Disagree-Strongly Agree)

| Items | |
| --- | --- |
| **Understanding of Health Information** | |
|  | 1. I can understand the instructions for medication provided by the hospital app. |
|  | 2. I can understand the emergency manual provided by health-related apps. |
|  | 3. I can understand the health check-up results provided by the hospital app. |
|  | 4. I can understand the terms of the privacy consent form when registering on the hospital app. |
|  | 5. I can understand the nutritional information of food provided by health-related apps. |
|  | 6. I am aware of the precautions for online payment when paying medical bills. |
|  | 7. I can understand the information about health check-ups (such as target, date, price, fasting requirements, etc.) provided by the hospital app. |
| **Utilization of Digital Devices** | |
|  | 8. I can save photos and texts about healthy activities found on the Internet. |
|  | 9. I can use the desired services (payment, location search, etc.) through the hospital kiosk. |
|  | 10. I can delete health-related apps that I have used. |
|  | 11. I can book and confirm medical services through the hospital app. |
|  | 12. I can find a suitable hospital for my symptoms using my smartphone. |
|  | 13. I can find information about disease symptoms and treatments using my smartphone. |
|  | 14. I can use the online “store” (e.g., Apple App Store or Google Play Store) on my device to find health-related apps. |
|  | 15. I can use appropriate words or search terms to find the health service information I want on the Internet. |
|  | 16. I can access hospital websites through an Internet search. |
|  | 17. In an emergency, I can find information about nearby hospitals using my smartphone. |
| **Use Intention** | |
|  | 18. I believe it is necessary to exchange health information online. |
|  | 19. I am willing to use health-related apps to collect health information. |
|  | 20. I have a lot of interest in health-related apps. |
|  | 21. I am interested in learning health knowledge or skills from the Internet. |
|  | 22. I find the necessity and convenience of health management through health-related apps. |
|  | 23. Using health-related apps improves my ability to manage my health. |
| **Utilization and Decision of Health Information** | |
|  | 24. I can judge whether the health information found on my smartphone is trustworthy. |
|  | 25. I can evaluate the pros and cons of various treatment methods provided by the hospital app. |
|  | 26. I can determine the medical services I need. |
|  | 27. I can judge how to use the health information provided by health-related apps. |
|  | 28. I can determine if the health information found on the Internet is written for commercial purposes (advertisements). |
|  | 29. I can use the health information provided by health-related apps for disease management. |
| **New Items** | |
|  | 30. I can register as a member on the hospital website or app. |
|  | 31. I can find my test results or prescription details on the hospital website or app. |
|  | 32. I can find information about certificate issuance and medical records on the hospital website or app. |
|  | 33. I can make online payments for medical bills through the hospital website or app. |
