# Supplementary material for: Measuring Digital Health Literacy in Older Adults: Development and Validation Study
Source: J Med Internet Res. 2025 Feb 5;27:e65492. doi: 10.2196/65492 (PMC11840366; doi:10.2196/65492)
Supplement: Multimedia Appendix 5 [file jmir_v27i1e65492_app5.docx]

**Appendix 5.** Digital Health Literacy Scale items after confirmatory factor analysis. 5-point Likert-scale. 0–4 (Strongly Disagree-Strongly Agree)

| Items | |
| --- | --- |
| **Utilization of Digital Devices** | |
|  | 1. I can access the hospital website through an Internet search. |
|  | 2. I can register as a member on the hospital website or app. |
|  | 3. I can delete health-related apps that I have used. |
|  | 4. In an emergency, I can find information about nearby hospitals using my smartphone. |
|  | 5. I can make online payments for medical bills through the hospital website or app. |
|  | 6. I can use the online "store" (e.g., Play Store or App Store) on my device to find health-related apps. |
|  | 7. I can use appropriate words or search terms to find the health service information I want on the Internet. |
|  | 8. I can use the desired services (payment, location search, etc.) through the hospital kiosk. |
|  | 9. I can book and confirm medical services through the hospital app. |
|  | 10. I can find my test results or prescription details on the hospital website or app. |
| **Understanding of Health Information** | |
|  | 11. I can understand the instructions for medication provided by health-related apps. |
|  | 12. I can understand the emergency manual provided by health-related apps. |
|  | 13. I can understand the nutritional information of food provided by health-related apps. |
|  | 14. I can understand the information about health check-ups (such as target, date, price, fasting requirements, etc.) provided by health-related apps. |
|  | 15. I can understand the health check-up results provided by health-related apps. |
| **Utilization and Decision of Health Information** | |
|  | 16. I can judge whether the health information found on my smartphone is trustworthy. |
|  | 17. I can evaluate the pros and cons of various treatment methods provided by health-related apps. |
|  | 18. I can determine if the health information found on the Internet is written for commercial purposes (advertisements). |
|  | 19. I can judge how to use the health information provided by health-related apps. |
|  | 20. I can determine the medical services I need. |
| **Use Intention** | |
|  | 21. I believe it is necessary to exchange health information online. |
|  | 22. I have a lot of interest in health-related apps. |
|  | 23. I am willing to use health-related apps to collect health information. |
|  | 24. I find the necessity and convenience of health management through health-related apps. |
|  | 25. Using health-related apps improves my ability to manage my health. |
